# Flow Dynamics and Pressure Modulation in a Patient-Specific Upper Airway using a Pulsating Nasal Jet

**DOI:** 10.21203/rs.3.rs-7303826/v1

**Published:** 2025-08-22

**Authors:** Muhammad Aseem, Elias Sundström, Liran Oren

**Affiliations:** University of Cincinnati; KTH Royal Institute of Technology, FLOW; University of Cincinnati

## Abstract

Pulsating airflow jets delivered via nasal cannula offer a promising, comfortable, non-invasive alternative to continuous positive airway pressure (CPAP) for treating obstructive sleep apnea (OSA). However, the fluid dynamic mechanisms by which pulsatile flow influences upper airway pressure remain poorly understood in anatomically realistic geometries. This study used large eddy simulations (LES) to examine pressure and flow characteristics of pulsating nasal jets within a patient-specific upper airway model. Two airflow conditions were simulated: (1) steady high-flow nasal cannula (HFNC) at 40 L/min and (2) pulsatile flow at 20 Hz with a 30% duty cycle, matched to the same mean flow rate. Each pulse generated a vortex ring that impinged on the nasal walls, creating localized high-pressure regions and asymmetric shear stress. Compared to steady flow, the pulsatile jet increased time-averaged pharyngeal pressure by up to 50%. Spectral analysis revealed that 20 Hz pressure oscillations were confined to the nasal cavity and pharynx, dissipating before reaching the lower airway. These effects, shaped by jet-wall interactions in complex anatomy, diverge from classical vortex dynamics. Pulsatile nasal flow may offer a precise, geometry-responsive method for upper airway stabilization and a more tolerable alternative to CPAP for OSA therapy.

## 1. Introduction

Obstructive sleep apnea (OSA) is a common sleep-related breathing disorder characterized by recurrent collapse of the upper airway during sleep.^1^ This condition affects millions worldwide and is associated with serious health risks due to intermittent hypoxia and sleep fragmentation. The current gold-standard treatment, continuous positive airway pressure (CPAP), pneumatically splints the pharyngeal airway open.^2,3^ CPAP is efficacious when used consistently, but patients often cite the discomfort related to the tight-fitting mask required by current devices as the main reason for discontinuing their therapy.^4,5^ High-flow nasal cannula (HFNC) therapy has emerged as a less invasive alternative that can deliver modest positive pressure.^6,7^ In this therapy, continuous flow is injected into the nares via a nasal prong design, which is generally more comfortable than CPAP masks due to the absence of a required facial seal. However, HFNC typically cannot provide the pressure levels required for OSA therapy to prevent airway collapse.^8^ This limitation has motivated the exploration of novel non-invasive therapies that can enhance upper airway patency without the need for a face mask.

One promising approach is the use of pulsating airflow jets delivered via nasal cannulas.^9^ Unlike steady flow, a pulsatile jet produces dynamic pressure oscillations and coherent vortex structures that interact with the airway walls in unique ways. Prior observations showed that pulsating airflow can achieve therapeutic pharyngeal pressures equivalent to or exceeding those of CPAP in awake patients. This study demonstrated that pulsating nasal airflow delivered via a nasal cannula produced peak pharyngeal pressures up to ~ 20 cmH_₂_O. These values are significantly higher than those from HFNC (maximum of 5.1 cmH_2_O^6,7^), and match the typical pressure levels required for OSA therapy.

It is well known that a starting jet or pulsed jets generate leading vortex rings that travel downstream. Upon impinging on a boundary, these rings can induce complex flow phenomena, including secondary vortices and localized pressure surges. Previous in vitro experiments^10–12^ involving vortex ring-wall interaction have shown that at sufficiently high Reynolds numbers, the primary ring expands, inducing boundary-layer separation that spawns secondary and even tertiary vortices. These interactions can cause rebound effects and eventual cascading flow breakdown. However, these studies were conducted in simplified geometries such as flat plates, inclined walls, or concave cavities, rather than anatomically realistic airways. For example, experiments with vortex rings impinging on inclined surfaces revealed asymmetric, helical vortex patterns.^11^ The portion of the ring hitting the wall first generates uneven vorticity and helix-like structures that migrate along the ring, away from the impact site. Similarly, vortex rings impacting confined concave cavities exhibit altered dynamics: increased vorticity at the cavity lip can disrupt the formation of classical secondary rings, and direct vortex impact on the cavity edge can even spawn additional vortex rings that travel in opposite directions.^10^ These prior works highlight how geometry and boundary conditions drastically influence vortex behavior. However, it remains unclear how such vortex–wall interaction principles translate to the upper airway, where the walls form a highly irregular, compliant passage.

Despite this promise, the mechanics of pulsatile jets in an anatomical airway remain incompletely understood. The upper airway has a complex geometry, characterized by narrow nasal passages, a sharp nasopharyngeal bend, and collapsible pharyngeal walls. How a train of vortex rings and jet bursts navigate this geometry, and how the resulting pressure field is distributed, is not obvious from the existing vortex ring literature. Using pulsating airflow as a therapeutic modality raises additional important questions: Do pulsatile jets predominantly create beneficial pressure elevations in the nose and pharynx? How quickly do the introduced vortex structures break down into turbulence in such a confined, winding airway? Are these large pressure oscillations transmitted into the lungs, or do they focus their effects more locally along the upper airways? The present study addresses these gaps by applying large-eddy simulations (LES) of pulsating nasal airflow in a patient-specific upper airway model. By integrating fluid dynamics insights from prior vortex ring experiments with state-of-the-art anatomical airflow modeling, we aim to elucidate the flow patterns and pressure oscillations generated by pulsating flow and to assess their potential to stabilize the airway for OSA therapy.

## 2. METHODOLOGY

### 2.1. Upper Airway Model Geometry

**Figure 1:**
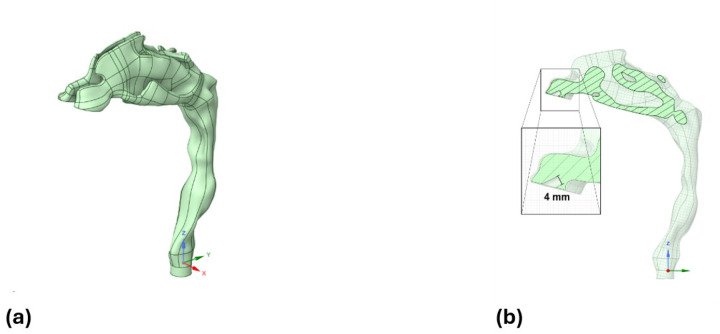
Patient-specific upper airway geometry reconstructed from CT imaging. (a) Isometric view showing the full computational domain from the nares (inlet) to the tracheal outlet below the larynx. b) Sagittal cross-section showing nasal prong insertion at ~45° relative to the hard palate. A zoomed inset highlights the 4 mm inner diameter of the nasal prongs used to model a high-flow nasal cannula interface.

A patient-specific upper airway geometry was reconstructed from an anonymized maxillofacial CT scan of a healthy adult. All methods were carried out in accordance with relevant guidelines and regulations. The study protocol was reviewed and approved by the University of Cincinnati Institutional Review Board, which determined that the use of fully de-identified CT images did not require informed consent. Each CT slice was 0.63 mm thick with an in-plane resolution of 0.43 mm/pixel. The airway lumen was segmented using the 3D Slicer software.^13^ The resulting 3D model (STL format) was refined in MeshLab^14^ and subsequently imported into ANSYS SpaceClaim to create a smooth solid CAD model. The model domain extends from the nares (nostril openings) at the inlet to the tracheal section just below the larynx (Fig. 1a). The model also included simulation of nasal prongs (4 mm inner diameter) that were inserted ~ 6 mm into each nostril at ~ 45° relative to the hard palate (Fig. 1b), approximately along the centerline to maximize jet penetration before impingement on the nasal wall. This geometric setup was designed to mimic a high-flow nasal cannula interface.

The airway volume was discretized using an unstructured tetrahedral mesh generated in ANSYS Meshing. We applied fine mesh refinement in the nasal vestibule and valve regions to capture steep velocity gradients. Five inflation layers (with a growth rate of 1.2) were added at the walls to resolve the boundary layer. The anterior nasal cavity, with its intricate geometry, was meshed at roughly twice the base resolution to capture the complex flow patterns within it better. The final mesh contained approximately 9.5 million elements.

### 2.2. Boundary Conditions

**Figure 2:**
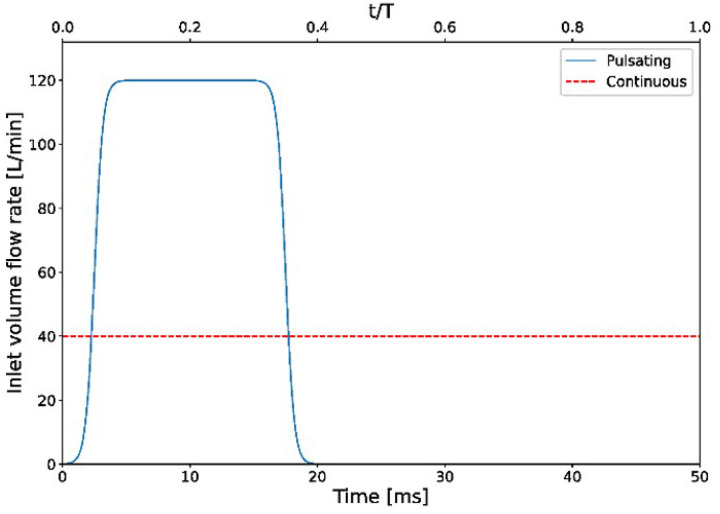
Inlet flow rate waveforms for the nasal cannula. The continuous case (dashed red line) is a steady 40 L/min flow. The pulsatile case (solid blue line) delivers the same 40 L/min mean flow via 20 Hz pulses with a 30% duty cycle (peaks of 120 L/min during each pulse).

Boundary conditions were designed to replicate a strong inspiratory effort combined with supplemental flow from nasal cannula prongs. A baseline inhalation flow of 30 L/min (approximate Reynolds number *Re ~* 2000 based on the nasal inlet diameter) was applied uniformly across the nares inlet plane, representing a normal-to-heavy peak inspiratory flow.^15^ Superimposed on this, we modeled two cases of uniformly-distributed nasal cannula airflow: (1) a continuous steady jet of 40 L/min (prong exit *Re ~* 8000), and (2) a pulsatile jet with a mean flow of 40 L/min delivered as 20 Hz bursts at 30% duty cycle (these parameters were selected following prior work demonstrating effective pressure generation at high-frequency oscillations^9^, and reflects a feasible operating range for pulsatile flow devices). In the pulsatile case, each “on” phase lasted 15 msec with a peak flow of 120 L/min (*Re ~*24000) during that interval, such that the time-averaged flow matched 40 L/min over a full cycle. Given the high *Re* and expected vortex-ring breakdown during jet impact, LES was used to resolve the unsteady turbulent structures within the pharyngeal region. Figure 2 illustrates the mass flow rate waveforms for the continuous and pulsatile injection. Boundary smoothing was applied with the pulsatile injection to model the waveform transitions. All walls were treated as no-slip boundaries, and a constant gauge pressure of 0 Pa (i.e., atmospheric) was imposed at the distal (tracheal) outlet.

### 2.3. Simulation Setup

**Figure 3:**
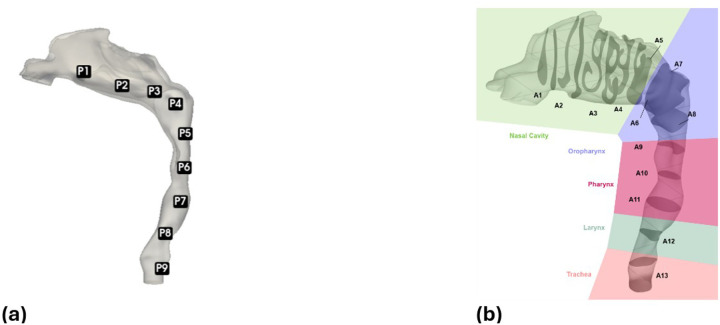
Locations of monitoring points (a) and cross-sectional data planes (b) defined along the airway model for analysis. A total of 9 points (centerline locations) and 13 perpendicular planes span the nasal cavity (P1–P3, A1–A5), oropharynx (P4, A7–A8), pharynx (P5–P7, A9–A11), larynx (P8, A12), and trachea (P9, A13).

All simulations were performed using ANSYS Fluent. Each case (continuous and pulsatile) was simulated for a physical duration of 0.1 s. For the continuous 40 L/min flow, this duration was sufficient to reach a quasi-steady state (the monitored flow variables became nearly constant in time). The pulsatile case covered two full pulse cycles (each 50 msec long) to observe periodic behavior. We used an implicit unsteady solver with a bounded second-order time integration scheme to accurately capture transient dynamics.^16^ LES was employed with the Wall-Adapting Local Eddy-viscosity (WALE) subgrid-scale model to resolve turbulent structures while accounting for near-wall effects. The time step was fixed at 2 × 10^−6^ s, which kept the convective Courant number below 1 in most of the jet region, ensuring numerical stability and accuracy. Flow field data (velocities and pressures) were recorded every 0.005 sec at specified monitoring points and cross-sectional planes throughout the domain (Fig. 3). Pressure–velocity coupling was handled with the PISO algorithm, chosen for its efficiency and stability in resolving fast transients.^17^ The computations were executed on the Ohio Supercomputer Center, using 140 CPU cores distributed across 5 nodes.

## 3. MODEL AND VERIFICATION

### 3.1. VALIDATION Comparison with Experiment

**Figure 4:**
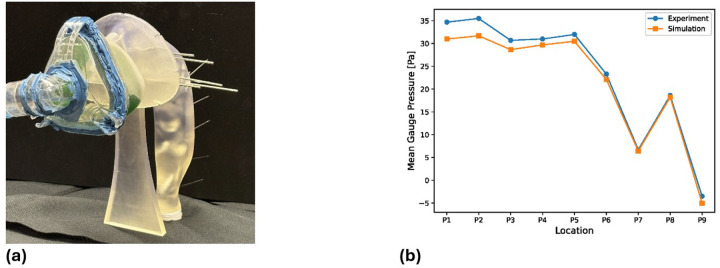
Experimental setup and pressure validation. a) 3D-printed airway model with wall pressure ports (P1–P9) for pressure measurements. b) Comparison of measured transmural pressure (points) vs. CFD-predicted pressure (line) along the airway for a steady 30 L/min inspiratory flow.

To validate the CFD model, we conducted an experimental measurement of airway pressures using a life-size replica of the same anatomy. The patient-specific airway was 3D-printed (stereolithography) and instrumented with 15 small pressure taps (1.6 mm diameter) along the airway wall (Fig. 4a). The pressure port locations were located on the walls at the same levels as in the simulations. Each port was connected to a pressure transducer (Honeywell FPG, 0–5 inches H_₂_O) and sampled at 1000 Hz for 1 s via a NI-9234 data acquisition system.

Replicating the exact combined flow condition of the simulation (simultaneous 30 L/min inspiration plus pulsation) was not feasible with our benchtop setup. Therefore, we performed the experiment under the baseline inhalation flow only. A constant 30 L/min airflow was delivered through a nasal mask attached to the model’s nares (Fig. 4a), matching the simulation’s inspiratory boundary condition. Figure 4b compares the measured transmural pressure (inside minus ambient) at each port with the pressure predicted by the CFD for the same 30 L/min steady inflow.

The simulation and experiment show good agreement in pressure distribution (Fig. 4b). The mean gauge pressure measured on the wall is compared with the predicted value at the monitoring point. The steady 30 L/min flow generated a slight positive pressure in the nasal cavities and nasopharynx, which then decreased through the pharyngeal-tracheal region. Both the measured and simulated pressure profiles exhibit a small pressure recovery at port P8, corresponding to a local airway expansion in the pharynx that converts kinetic energy back into pressure. The largest discrepancy was approximately 11.9% at one of the nasal cavity ports (P2), but overall, the absolute mean error between simulation and experiment was only 4.63%. This level of accuracy gives confidence that the CFD model can reliably capture pressure behavior in the upper airway under the given flow conditions.

### 3.2. Grid Sensitivity

A mesh sensitivity study was conducted using four progressively refined unstructured grids containing approximately 6M, 8M, 9.5M, and 12M cells. The predicted pressure fields were compared across these grids. We found that increasing the mesh from 9.5M to 12M cells changed the pressures, time-averaged across the length of the pulse and area-averaged across each cross-section by less than 2%. Additionally, no discernible changes were observed in the evolution of the vortex ring structures or wall shear stress distributions between these two meshes. Based on this convergence in both integral and structural flow metrics, the 9.5M cell mesh was deemed sufficient for all subsequent simulations.

### 3.3. Energy Spectral Analysis

**Figure 5:**
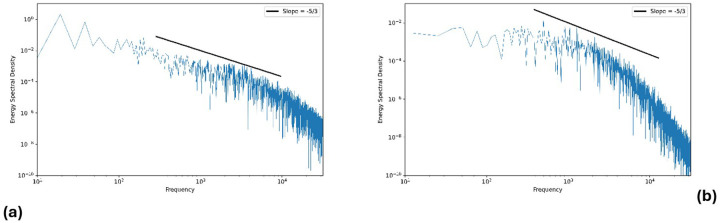
Energy spectral density of velocity fluctuations at a nasopharyngeal monitoring point (P4). The spectrum follows an approximate K^−5/3^ slope (solid black line) in the mid-frequency range, consistent with Kolmogorov’s inertial subrange and indicating adequate turbulence resolution by the LES model. a) Pulsating flow. b) Continuous flow

As an additional verification of the LES solution quality, we examined the turbulent kinetic energy spectrum at a representative point in the flow. Specifically, we computed the velocity fluctuation spectrum at a monitoring point located in the nasopharynx (P4). The spectrum was obtained by performing a Fast Fourier Transform on a time series of velocity fluctuations sampled at 500 kHz over 10.8 msec. The resulting spectrum (Fig. 5) demonstrates an approximate − 5/3 slope across roughly one decade in the mid-frequency range, indicating the presence of a well-resolved inertial subrange. This suggests that our mesh and time-step were fine enough to capture the energy cascade from large to smaller eddies without excessive dissipation at the resolved scales. Figure 5a corresponds to pulsatile flow and Fig. 5b to continuous flow.

## 4. RESULTS

### 4.1. Opening Vortex Formation at Pulse Onset

**Figure 6:**
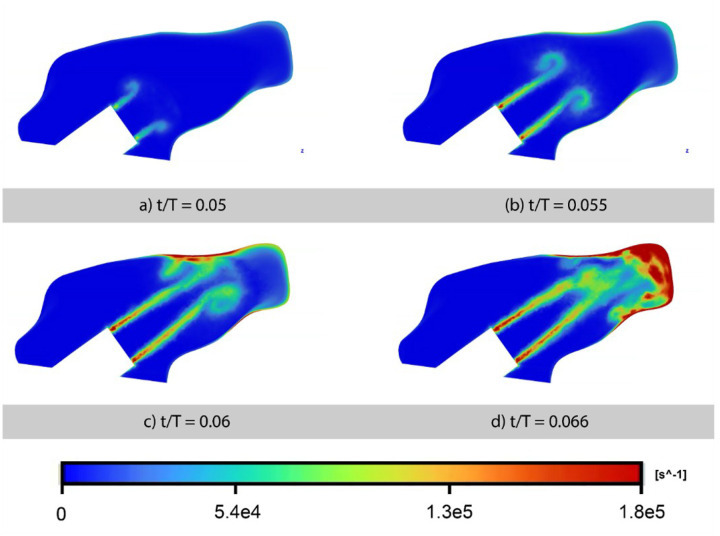
Vorticity contour snapshots at the onset of a pulsatile jet flow, illustrating the formation and advection of an “opening vortex” ring just downstream of the nasal prong. Times shown correspond to (a) 0.050, (b) 0.055, (c) 0.060, and (d) 0.066 of the pulse cycle (t/T). The vortex forms near the prong exit and travels into the nasal cavity, temporarily elevating local pressure before impinging on the nasal wall.

At the start of each pulsation cycle, the sudden jet of airflow generates a distinctive vortex structure within the nasal cavity. Figure 6 shows vorticity contours in a sagittal slice through the nasal passage during the initial milliseconds of the pulse. By time *t/T* = 0.050 (where *T* is the 50-msec pulse period), a coherent vortex ring begins to form just downstream of the nasal prong exit. This structure is characterized by a concentrated region of high vorticity and a well-defined core, corresponding to a local increase in static pressure resulting from induced pressure and local momentum deceleration. As the pulse continues to *t/T* = 0.055, the vortex detaches from the jet and convects downstream along the airway. By *t/T* = 0.060, the vortex is fully developed and moving further downstream. In our model, the vortex eventually impinges on the nasal vestibule wall by around *t/T* = 0.066 (Fig. 6d). While the exact timing and location of vortex impingement would likely vary with different prong insertion angles or patient anatomies, the general phenomenon of a pulse-generated vortex is expected to occur in pulsatile nasal flows. This “opening vortex” is analogous to the starting vortex ring produced by a pulsed free jet, which typically generates an initial pressure surge upon formation. However, in the confined nasal cavity, this vortex quickly impinges on the walls, altering its development as described.

### 4.2. Spatial Velocity Distribution During Mid-On-Pulse

**Figure 7:**
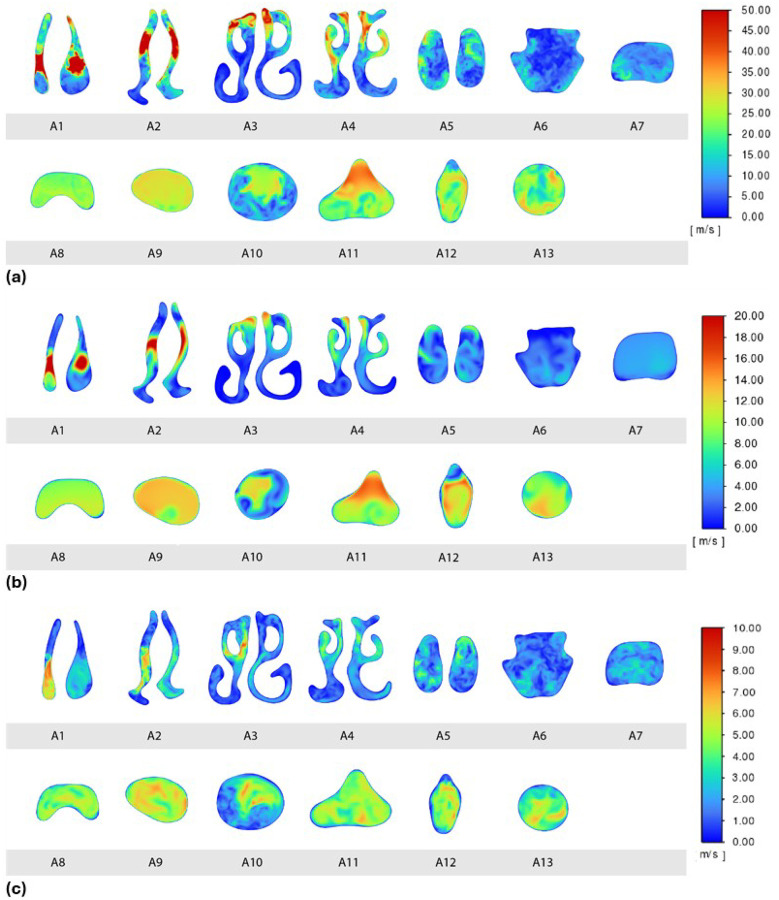
Cross-sectional velocity magnitude contours under different conditions. a) Pulsatile flow at mid-on-pulse (t/T = 0.15). b) Continuous flow at 40 L/min. c) Pulsatile flow at mid-off-pulse (t/T = 0.65). The pulsatile case exhibits similar flow patterns to the continuous case, but with much higher peak velocities during the active pulse and markedly lower velocities during the no-flow interval compared to the steady case.

At mid-pulse (*t/T* = 0.15), the airflow velocity field shows complex spatial patterns through the airway. Figure 7a presents velocity magnitude contours on a series of cross-sectional slices (A1–A13) spanning from the nasal vestibule to the trachea. In the anterior nasal cavity (slices A1–A3), the pulsatile jet creates narrow regions of very high velocity adjacent to regions of low velocity, reflecting the jet’s interaction with the nasal walls and the previously described opening vortex. At slices A1 and A2, these high velocity magnitude regions trace the jet trailing the vortex, and at A3 the vortex impinges on the superior nasal cavity wall, causing a localized peak in velocity. As the airflow progresses posteriorly, it decelerates upon entering the wider nasopharynx (A5) and slows further in the oropharynx (A6–A8) due to increased cross-sectional area. After passing through the constricted laryngeal region (around slice A12), the flow re-accelerates slightly into a jet-like profile in the pharynx (A10–A11) but then slows again in the trachea (A13). The sudden expansion beyond the larynx leads to flow separation from the wall and a recirculation zone forming in the upper trachea, as indicated by reversed or low-velocity flow near the airway walls in those downstream slices.

When compared to the continuous flow case (Fig. 7b), the spatial distribution of velocities is qualitatively similar – the high-speed regions occur in roughly the same locations along the airway for a given mean flow rate. However, the magnitudes differ markedly. The continuous 40 L/min jet produces much lower peak velocities than the pulsatile jet. In other words, with both cases delivering the same mean flow, the pulsating injection results in much higher instantaneous velocities during the pulse than the steady flow does at any time.

### 4.3. Spatial Velocity Distribution During Mid-Off-Pulse Phase

During the pulsatile flow’s off phase (mid-shut-off, *t/T* = 0.65), the flow distribution changes significantly in the nasal region. With no jet being injected from the prongs during this phase, the only airflow arises from the baseline inspiratory flow of 30 L/min entering through the nares. Thus, velocity magnitudes throughout the nasal cavity are substantially lower than during the pulse-on phase (Fig. 7a vs. Figure 7c). In fact, the pattern in Fig. 7c represents a normal peak inspiratory flow distribution without any (pulsatile or continuous) flow augmentation. Downstream of the nasal cavity, the contours in the oropharynx, pharynx, and trachea remain qualitatively similar in shape to those observed during midpulse. The airflow still accelerates through narrower sections and decelerates in expansions, but all velocity values are much smaller without the pulsatile boost. Essentially, the pulse-off phase resembles a low-speed version of the flow, confirming that the added jet is responsible for the high-velocity peaks observed during the on-phase.

### 4.4. Pressure Distribution

**Figure 8:**
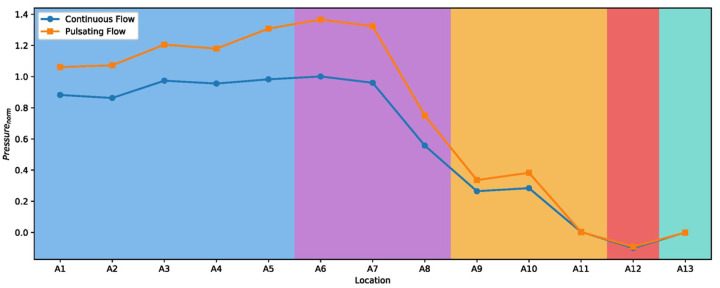
Normalized mean pressure distribution along the airway for pulsatile versus continuous flow. Pressures are normalized to the peak mean pressure of the continuous flow case. The pulsatile flow maintains a higher pressure than the continuous flow throughout the airway, with the largest differences occurring in the pharyngeal region.

A primary goal of introducing pulsating jets is to further increase the mean airway pressure beyond the elevated levels already achieved with HFNC. The mean pressure distribution along the airway model was calculated for each of the data planes (Fig. 8). To emphasize the difference between cases, all pressure values were normalized by the maximum pressure value calculated in the continuous flow case (predicted to occur in the oropharynx). In the pulsatile case, “mean” refers to the time-averaged pressure over a full cycle, allowing for direct comparison with the steady case.

Both the continuous and pulsatile flows exhibit a notable pressure drop from the nasal cavity into the oropharynx. This can be explained by the flow convergence and acceleration in that region: the two streams from the left and right nasal passages combine into one nasopharyngeal airway, whose cross-sectional area is less than the sum of the two nasal cavities. By the continuity principle, the airflow must speed up as it passes through this bottleneck, and according to Bernoulli’s equation, a higher velocity corresponds to a lower static pressure. Additionally, the airway path makes a turn (approximately 90°) as it transitions from the horizontal level of the nasal cavity into the vertical level of the oropharynx. This change in flow direction causes a loss of momentum, further contributing to the pressure drop between the nasopharynx and oropharynx (specifically, between A7 and A8). The pressure continues to decline slightly through the pharynx, then beyond the pharyngeal region, it levels off. From the open larynx into the trachea, the cross-sectional area remains fairly constant, and thus the pressure remains relatively uniform. This overall pressure distribution pattern underscores the dominant role of airway geometry in shaping where pressure losses occur.

### 4.5. Wall Shear Stress

**Figure 9:**
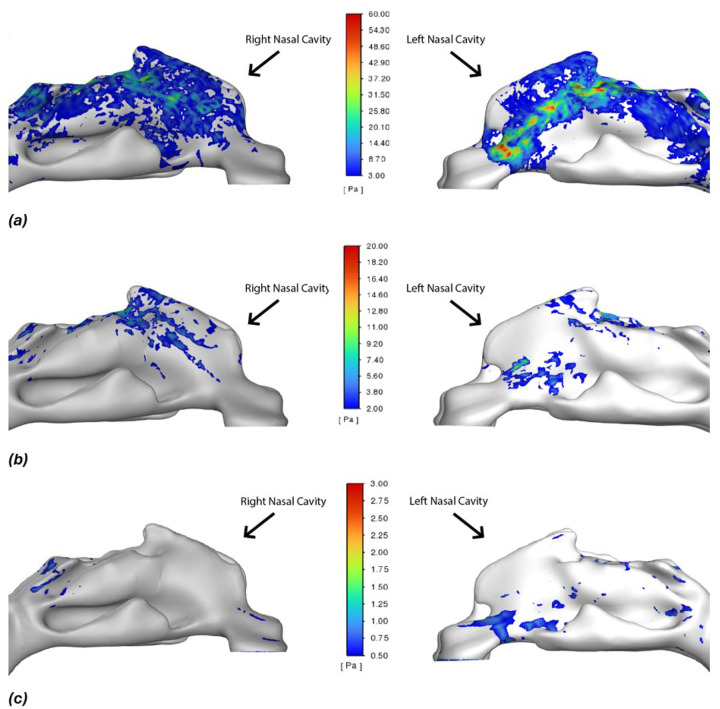
Wall shear stress (WSS) contours on the airway walls. a) Pulsatile flow at mid-on-pulse. b) Continuous flow. c) Pulsatile flow at mid-off-pulse Phases shown are the same as in Fig. 7. The left nasal cavity (arrow) experiences a much higher shear stress during the pulsatile on-phase (a) compared to continuous flow (b), whereas the right cavity remains low-shear. These asymmetric patterns underscore the influence of individual anatomy on shear distributions.

Wall shear stress (WSS) quantifies the drag force of the airflow acting on airway surface and is a key determinant of mucosal stimulation, potential tissue irritation, and patient comfort. The WSS is mapped throughout the airway for both continuous and pulsatile flows (Fig. 9). A striking feature is the left–right asymmetry: due to the patient-specific anatomy in this case, the left nasal passage experiences much higher shear stress than the right side. In this individual, the left nasal cavity’s airflow is directed toward the olfactory region, causing elevated shear in that area, whereas the right cavity has a more open path that produces relatively little shear on the walls. Beyond the nasal cavity, WSS levels drop off dramatically; in most of the pharynx, larynx, and trachea, the shear stress is near zero because the airflow core is separated from the walls. This left–right disparity in wall impact is reminiscent of a vortex ring striking an inclined surface, where the near-wall side of the ring generates stronger vorticity and more complex flow than the far side.

When comparing flow modes, the pulsatile jet’s impact on shear stress is evident. Under continuous flow (Fig. 9b), the maximum shear stress on the left nasal wall is moderate. But during the pulsatile flow’s active phase (Fig. 9a), the peak WSS on that same wall is roughly three times higher. The pulsation drives a stronger jet against the nasal wall on one side, amplifying shear stress there. During the pulsatile off-phase (Fig. 9c), the shear pattern resembles the continuous case (since only the base inspiratory flow is present). The right nasal cavity remains low-shear in all scenarios.

### 4.6. Spectral Analysis

**Figure 10:**
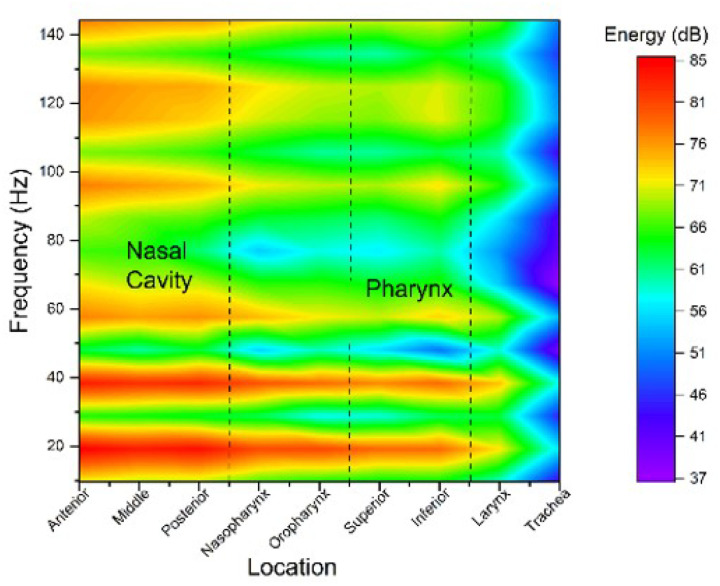
Pressure spectral density distribution along the airway model. The 20 Hz fundamental frequency (injected pulse rate) is prominent, but its relative amplitude decays along the airway, with more energy appearing at higher frequencies, indicating that the pulsating flow loses its coherence and becomes increasingly turbulent.

The frequency content of pressure fluctuations at 13 cross-sectional planes along the model (Fig. 10) was analyzed to understand how the pulsation propagates and dissipates through the airway. A spatial contour plot, similar to a spectrogram, was created from the pressure spectra computed at each plane using FFT on time-resolved pressure signals. At the nasal inlet, the spectrum exhibits a dominant peak at 20 Hz, which is the driving frequency of the pulsating jet. This 20 Hz component gradually diminishes in strength downstream. By the time the flow reaches the end of the oropharynx, the amplitude of the 20 Hz peak is significantly reduced. Below the larynx, the 20 Hz signal is barely discernible above the background. Instead, higher-frequency components become prominent in these distal regions, indicating a transition from coherent pulsation to broadband turbulent fluctuations.

This trend suggests that the coherent pulsation imposed by the device is largely absorbed or broken down by the complex upper airway. While the initial pulse injects energy at a discrete frequency of 20 Hz, the flow encounters the complex, curved geometry and transitions to turbulence; much of that energy is redistributed across a wider spectrum, including smaller-scale, higher-frequency eddies. The oropharynx appears to be a key region where the organized pulsation loses coherence, likely due to flow separation and vortex dissipation there. Consequently, the 20 Hz oscillation is essentially absorbed in the upper airway, with negligible transmission to the lower airway. This rapid breakdown of the coherent pulsation is consistent with prior observations of vortex rings dissipating into turbulence after wall impact, and it underscores that the pulsatile energy remains localized to the target region (nasal–pharyngeal airway).

## 5. DISCUSSION

### Comparison to Prior Vortex Ring Studies:

The current results show that each pulse generates a pronounced vortex ring (an “opening vortex”) in the nasal cavity, which subsequently impinges on the airway walls. This behavior is highly consistent with prior observations of vortex rings formed by starting jets.^18^ In the simulations, the nascent vortex ring travels only a short distance from the prong’s exit before striking the nasal vestibule wall (within ~ 0.06 s of the pulse onset). In an idealized setting (e.g., a vortex ring impinging on a flat or inclined plate), one would expect the ring to induce a wall boundary layer and possibly form a secondary vortex ring from the shed vorticity. Indeed, previous experiments have documented that when a vortex ring impacts a surface at normal incidence, a sheet of opposite-sign vorticity is generated on the wall, which rolls up into a secondary ring, causing the primary ring to slow and “rebound” away from the wall.^11^ In our anatomical scenario, the same fundamental process likely begins (the pulse-generated vortex creates a region of wall vorticity), but the outcome diverges due to geometric complexity. The confined nasal cavity does not provide the flat, open surface needed for a symmetric secondary vortex ring to fully develop. Instead, the primary vortex’s collision with the uneven nasal walls almost immediately disrupts its coherent structure. This is analogous to the high-confinement cases reported by Ahmed and Erath^10^, where the vortex ring’s interaction with a cavity lip generated intense vorticity that halted the classical secondary ring formation. In our simulations, we did not observe a clear secondary vortex ring separating and orbiting the primary ring. This is likely because any nascent secondary vortex is quickly absorbed into the complex, turbulent flow that ensues in the nasal passage. Notably, the vortex did not exhibit a pronounced rebound as seen in simpler vortex–wall studies, which again can be attributed to the anatomical channel “capturing” the vortex rather than allowing it to ricochet. These differences highlight how anatomical confinement alters the dynamics of the vortex ring: the upper airway’s irregular geometry essentially short-circuits the neat sequence of secondary and tertiary ring formation and rebound that occurs for rings impinging on simpler surfaces.

Despite the altered progression, our findings remain consistent with the physics of vortex–boundary interactions as described in prior studies. For instance, the vortex impingement in the nasal cavity produced transient high pressures at the contact region (Fig. 6d), consistent with the notion that a vortex ring approaching a wall causes an initial stagnation pressure rise at the impact point. Furthermore, the flow immediately after impingement became highly three-dimensional and asymmetric, as the left and right nasal passages exhibited different vortex behavior (one side experienced a stronger jet impact and higher wall shear, as discussed below). This observation aligns with findings from inclined-wall collisions of vortex rings, where one side of the ring interacts more strongly, resulting in unevenly distributed vorticity. *Lim (1989)* documented, via dye visualization, that an inclined impact causes the near-wall side of the ring to form helical vortex filaments that convect away from the wall, eventually ejecting fluid radially in the symmetry plane.^12^ In our case, the patient-specific anatomy inherently creates an “inclined” or uneven impingement – for example, the left nasal passage had a sharper turn and narrower channel, leading to a more forceful vortex impact on the left lateral wall. As a result, we observed significantly higher wall shear stress on the left side of the nasal vestibule compared to the right (Fig. 9a vs 9b), an asymmetry directly attributable to the flow–structure interplay. This is qualitatively similar to prior vortex ring experiments at oblique angles, which found that the portion of the ring hitting first produces more intense local vorticity and a decidedly asymmetric flow pattern. Thus, even though the anatomical airway yields a very complex flow, the underlying phenomena of vortex generation, wall vorticity shedding, and asymmetric vortex stretching are grounded in classical vortex dynamics. Our study extends those dynamics to a realistic geometry, providing a bridge between fundamental vortex ring physics and the behavior of pulsed jets in the human upper airway.

### Pressure Oscillations and OSA Therapeutic Implications

A key finding of this work is that the beneficial pressure oscillations produced by the pulsatile flow are largely confined to the upper airway (nasal cavity and pharynx). The frequency analysis (Fig. 10) shows that the imposed 20 Hz pulsation is strong in the nasal region, but its amplitude decays progressively as the flow moves downstream; by the time airflow reaches the larynx and trachea, the 20 Hz component is almost entirely dissipated into broadband turbulence. In other words, the pulsatile jet delivers oscillatory pressure energy to the nose and throat, but very little of that organized oscillation penetrates the lower airway. This outcome is highly desirable for treating OSA. The pharyngeal airway is the segment that requires stenting pressure to prevent collapse, whereas the lungs and distal airways do not benefit from (and could be disturbed by) large alveolar pressure swings. By confining the oscillatory pressures to the upper airway, the pulsatile airflow targets the therapeutic effect where it is needed (to splint the collapsible airway) without subjecting the lower airways to strong pressure fluctuations. This is in contrast to high-frequency oscillatory ventilation, for example, which intentionally transmits oscillations to the lungs for gas exchange. In our application, we specifically *do not* want to oscillate the alveoli. Our LES results suggest that the complex nasopharyngeal geometry acts as a filter, rapidly absorbing and dispersing the pulsation energy. As the jet’s vortical structures interact with the narrow passages and sharp bend into the oropharynx, vortex breakdown and broadband turbulence emerge, c.f. Figure 10. This effectively damps the 20 Hz component beyond the pharynx. The behavior is consistent with established vortex dynamics, where coherent vortex rings lose coherence and dissipate their energy upon interacting with boundaries.^10,19^ Here, the *upper airway serves as a boundary-rich environment* that dissipates pulsatile energy before it can reach the lungs. Clinically, this means a pulsating nasal airflow device could provide oscillating positive pressure to splint the pharyngeal region (where OSA collapse occurs) while delivering a relatively steady flow to the lungs.

Another important observation is that the mean pressure elevations achieved with pulsatile flow were higher in the pharyngeal region than those from an equivalent steady flow (i.e., HFNC). Our simulations showed that, for the same mean flow rate, the time-averaged pressure in the pharynx was up to 50% higher with the 20 Hz pulsed jet than with continuous flow (Fig. 8). This corroborates recent patient measurements by Oren et al., who reported that pulsating airflow via nasal cannula attained pharyngeal pressures up to 20 cmH₂O, significantly exceeding what a continuous high-flow could produce. The mechanism for this pressure boost in our model is tied to the transient jet dynamics: during each pulse “on” phase, a surge of flow impinges on the airway walls, locally augmenting the pressure, and these surges occur repeatedly at 20 Hz. The pharyngeal airway, being a collapsible tube, benefits from even a brief high-pressure pulse because it resists collapse at that moment. The pulsatile nature effectively supplies a series of mini-CPAP breaths each second. Meanwhile, between pulses, pressure dips closer to baseline; however, the airway does not immediately collapse in these brief intervals, and the next pulse arrives to reinforce the stent.

Our discussion thus far suggests that pulsating jet airflow marries two beneficial aspects: (1) a higher peak pharyngeal pressure (for better splinting) and (2) localization of pressure oscillations to the upper airway (to avoid unnecessary lung stress). These features provide pulsatile nasal airflow with a promising clinical profile for OSA therapy, addressing some limitations of both CPAP (the need for a sealed interface to pressurize the airway) and HFNC (insufficient pressure support).

### Advancing Understanding of Jet–Wall and Vortex–Structure Interactions:

Beyond the clinical implications, this study provides new insights into vortex–structure interactions in an anatomical geometry. Previous vortex ring experiments have typically been conducted in clean setups (straight nozzles, flat, smoothly curved targets, or inclined surfaces) to isolate canonical flow behaviors. In reality, the upper airway presents a series of angled surfaces, curvatures, and bifurcating passages that significantly influence vortex evolution. Our results demonstrate how classical phenomena adapt in this setting. For example, one well-known effect in vortex ring impacts is that vortex rings *increase in diameter and slow down* as they approach a wall, with the core vorticity intensifying due to stretching. We qualitatively observed the initial vortex in the nasal cavity behaving in line with this: it formed near the prong (small diameter), then grew in size and slowed as it moved into the wider nasal space, indicating vortex stretching and interaction with surrounding walls. However, this coherence is short-lived, almost immediately, the ring encounters geometric confinement between the septum and the lateral wall, which alters its trajectory and structure. Our simulation captured the *early stages* of secondary vorticity development as the vortex hit the vestibule (manifesting as a shear layer on the wall in Fig. 6d), but the subsequent evolution of that vorticity was swept into the general turbulent flow. From an engineering perspective, this suggests that jet-wall interaction in a tortuous geometry is a highly efficient vortex “breaker.” Each pulse’s coherent structure is rapidly shattered into smaller eddies by successive encounters with anatomical structures (turbinates, airway curvature, etc.). The outcome is a quick transition to a complex, three-dimensional flow field that nonetheless carries the imprint of the jet pulses in its pressure distribution. We also gained insight into how asymmetry in geometry (differences between the left and right nasal passages) can lead to markedly different flow splits and wall impacts, something that fundamental studies with symmetric setups did not address. The left passage in our model functioned almost like a slightly more inclined or constricted impingement scenario, yielding higher shear and a more energetic impingement vortex, whereas the right side was more open and benign. This emphasizes that patient-specific anatomy can profoundly influence jet delivery and should be considered in designing pulsatile airflow therapies.

Finally, the study contributes to understanding jet–wall interactions under pulsatile conditions. Prior research have shown that pulsating jets can enhance surface interactions, for example, increasing heat transfer or erosion by repeated vortex impingement.^20–22^ In our simulations, pulsation similarly amplifies wall interaction in the nasal cavity. During the pulse-on phase, WSS (Fig. 9) was much higher than under steady flow, indicating more vigorous wall jets and recirculation induced by the vortex rings. While excessive shear stress may raise concerns about nasal comfort or mucosal health, the peak shear stress levels remained localized within physiologically tolerable limits (on the order of a few Pa). Interestingly, these elevated shear stress zones could offer secondary benefits, such as promoting mucociliary clearance or reducing stagnant zones in the nasal passages, analogous to how oscillatory airflow is sometimes used in chest physiotherapy to mobilize secretions.^23^ This remains speculative, but it highlights the multifaceted nature of pulsatile jet flows. The present simulations, focused on flow physics, lay the groundwork for future studies to explore such bioengineering aspects.

## 6. CONCLUSION

In summary, this work has demonstrated that pulsating nasal airflow generates complex vortex-driven flows that effectively elevate pharyngeal pressure while confining oscillatory energy to the upper airway. The results compare favorably with prior vortex ring studies, extending the knowledge of classical vortex-wall interactions into a biomedical setting. From a clinical biomechanics standpoint, the introduction of vortex pulses into the nasal airway emerges as a powerful mechanism to stabilize the collapsible pharynx in OSA. From an engineering standpoint, the study advances our understanding of how pulsed jets behave in non-ideal, convoluted domains – a step beyond canonical fluid mechanics problems. Future investigations will be needed to examine different pulse frequencies, waveforms, patient-specific anatomical variations, and wall compliance (moving) airway walls, to better understand and optimize performance across patients. Nevertheless, the insights gained here confirm that leveraging vortex dynamics in an anatomical flow can offer a compelling new avenue for non-invasive respiratory support. By marrying the clinical and engineering perspectives, we gain confidence that (vortical) pulsatile airflow can be tuned to maximize upper airway pressure benefits, minimize adverse effects, and ultimately improve therapy for OSA and related conditions.

## Data Availability

All data that support the findings of this study are available from the corresponding author upon reasonable request.
